# What is "biological Alzheimer's disease"?

**DOI:** 10.1590/1980-5764-DN-2024-E001

**Published:** 2024-06-10

**Affiliations:** 1 Universidade de São Paulo, Faculdade de Medicina, São Paulo SP, Brazil.

Almost 50 years ago, since Robert Katzman's article entitled "The Prevalence and Malignancy of Alzheimer's Disease: A Major Cause of Death", we have explained to society as a whole that Alzheimer's disease (AD) is the leading cause of dementia and a very serious illness^1^.

Katzman was referring to dementia due to AD. This is what everyone still understands as AD: a "synonym" for dementia.

It is now proposed that almost the same designation be given to a different condition: "biological Alzheimer's disease"^2,3^.

In many chronic conditions, the disease begins months, years, or even decades before clinical manifestation. Thus, it is possible to diagnose the disease before the appearance of any clinical signs or symptoms, as long as biological markers of the disease (i.e.: biomarkers) are available. This method has been and will continue to be very important in many diseases such as diabetes mellitus, coronary artery disease, cerebrovascular disease, and mainly cancer.

Since the first attempts to define biological AD *in vivo*, biomarkers in cerebrospinal fluid (CSF) and neuroimaging have been used^2,4^. The AT(N) framework (A for amyloid, T for phospho-Tau, and N for neurodegeneration) was initially proposed for research rather than routine clinical care, and it has been described as agnostic to clinical symptoms^2,4^. Now, with the development of plasma biomarkers, testing for biological AD became easier.

In the last Alzheimer's Association International Conference in July 2023, the diagnosis of biological AD was proposed to be used in clinical practice^3^. This proposal was based on the conception of a continuum from biomarker abnormalities to subjective decline, to mild cognitive impairment and then to mild dementia progressing to moderate, followed by severe dementia^3^. According to that proposal, biomarker abnormalities in the CSF, or amyloid-PET and/or Tau-PET, or plasma biomarkers abnormalities, particularly of beta-amyloid protein and phospho-tau protein, even in the absence of any clinical symptom, should be diagnosed as AD or as biological AD^3^. The adjective "biological" really does not change much of the meaning of the diagnosis for the patient or the lay public. For research it is a sound proposal.

The strongest opposition to the use of this concept in clinical practice came from the International Working Group, which holds that "biomarker-positive cognitively unimpaired individuals should be considered only at-risk for progression to Alzheimer's disease"^5^. It is clear that the meaning of words (or nomenclature or semantics) is the problem here.

In October 2023, after listening to criticisms and suggestions, the Alzheimer's Association Working Group took a step back and slightly modified its proposal^6^. In the draft entitled Revised Criteria for Diagnosis and Staging of Alzheimer's Disease: Alzheimer's Association Workgroup, it is clearly stated that these criteria "are not intended to be specific clinical practice guidelines, but rather criteria to inform diagnosis and staging of AD that reflect current science". It is also emphasized that routine diagnostic testing for asymptomatic individuals is not recommended, at this time^6^.

But as a clinical neurologist, I am very concerned about the lack of distinction between the diagnoses of biological AD and clinical AD.

This possible example should be considered: a 65-year-old man, with very mild complaints of memory decline, was diagnosed with biological AD after abnormalities were found in plasma biomarkers; then, he was referred to a neurologist. After neuropsychological examination, the diagnosis was subjective cognitive decline (SCD), meaning normal performance in cognitive evaluation. (Plasma biomarkers for AD should not have been requested for this patient with SCD^7^; but now it is too late). What should be the recommendation of the neurologist? Should this man leave his high position in a company, or stop running for the Senate or even for the Presidency? At least one study has shown that many individuals with SCD and biological AD do not progress to dementia or even to MCI after reasonably long intervals^8^. It has also been shown that the biological stage of AD was very important for the evolution of the clinical syndrome^8^. The biological stage of AD may be inferred by the presence of neurodegeneration in neuroimaging studies, and also from Tau-PET, which may be only positive in the medial temporal lobe, or may also include the temporal neocortex, meaning progression of biological AD^8^.

In the case described above, confirmatory CSF tests should be performed. If biological AD is confirmed, neuroimaging studies are needed to better define the stage of the disease. The decision of whether or not to use the recent approved treatments depends on how much the neurologist is convinced of their efficacy in very early AD (for which there are no studies, but theoretical support), also after explaining and listening to the patient's opinion.

Then, it is time to discuss with the patient his decision about continuing his career (which, in my opinion, should not be interrupted). However, if the press is notified of the AD diagnosis, the chance of a successful career will be destroyed, unless the lay public has been correctly informed about the new meaning of AD. To apply this new concept in clinical practice, there is a need for massive information for doctors and the lay public before using it.

AD is no longer synonymous with dementia. Biological AD is a risk factor for cognitive decline, which may take years to manifest or even never manifest clinically.

This is a critical issue.
